# Evaluating automated pollen monitoring for thunderstorm asthma and public health forecasting: a comparative study in Melbourne, Australia

**DOI:** 10.1007/s10661-026-16017-2

**Published:** 2026-10-13

**Authors:** Edwin R. Lampugnani, Usha Nattala, Danny Csutoros, Kale Sniderman, Beau Picking, Kathryn M. Emmerson, Elias Graf, Yanick Zeder, Erny Niederberger, Ed Newbigin

**Affiliations:** 1 Department of Medicine (RMH), University of Melbourne, Parkville, VIC 3010 Australia; 2 AirHealth Pty Ltd., PO Box 38, Parkville, VIC 3052 Australia; 3 School of Health Sciences, University of Melbourne, Parkville, VIC 3010 Australia; 4 Menzies Institute for Medical Research, College of Health and Medicine, University of Tasmania, Hobart, TAS 7001 Australia; 5 Melbourne Data Analytics Platform, University of Melbourne, Parkville, VIC 3010 Australia; 6 Victorian Department of Health, Melbourne, VIC 3000 Australia; 7 School of Geography, Earth and Atmospheric Sciences, University of Melbourne, Parkville, VIC 3010 Australia; 8 CSIRO Environment, Aspendale, VIC 3195 Australia; 9 Swisens AG, Meierhofstrasse 5a, 6032 Emmen, Switzerland

**Keywords:** Aerobiology, Bioaerosols, Exposure assessment, Hirst trap, Poaceae, Thunderstorm asthma

## Abstract

High-quality pollen measurements are essential for effective environmental monitoring and reliable public health forecasting. Automated pollen counters offer continuous, high-resolution observations that have the potential to reduce reliance on labour-intensive manual microscopy, but their performance must be evaluated against established methods. This study evaluates the performance of the SwisensPoleno Mars by comparing its daily and sub-daily pollen measurements of total pollen, and the two dominant airborne pollen types in Melbourne, grass and cypress, with those from a co-located Burkard Hirst-type spore trap at the University of Melbourne over the 92-day spring monitoring period in 2022. Data from the automated system showed a strong linear relationship with the co-located manual trap, with coefficients of determination (*R*^2^) of 0.92 for total daily pollen counts, 0.92 for grass pollen, and 0.86 for cypress pollen. Grass pollen measurements, which are central to epidemic thunderstorm asthma (ETSA) risk forecasting in Victoria, showed particularly high correspondence, including consistent classification into low, moderate, and high operational thresholds. The SwisensPoleno Mars also reproduced expected diurnal variation in pollen concentrations, demonstrating its ability to capture short-term changes in airborne pollen levels. These results indicate that automated pollen counters can deliver reliable observations at both daily and sub-daily scales while substantially reducing labour demands. The strong performance of the automated system supports its use for routine pollen monitoring, its suitability for supplementing or replacing manual counting in Victoria and its value and suitability for applications that benefit from high temporal resolution, including ETSA risk forecasting.

**Supplementary Information:**

The online version contains supplementary material available at https://doi.org/10.1007/s10661-026-16017-2.

## Introduction

Asthma is a common chronic respiratory condition in Australia that significantly affects individuals’ quality of life and places a considerable strain on government healthcare resources due to its high prevalence and associated morbidity (Australian Institute of Health and Welfare [AIHW], 2026; Shin et al., 2023). In the Australian state of Victoria, asthma-related hospital admissions show a clear seasonal pattern with admissions during the springtime peak partly driven by sporadic episodes of epidemic thunderstorm asthma (ETSA) (Silver et al., 2018; Thien et al., 2018). These high-impact events are of serious public health concern given the potential for very large surges in asthma cases, which can strain and possibly overwhelm healthcare systems (emergency call service, prehospital ambulance care and emergency departments) particularly in urban areas.

In response to the world’s largest ETSA event which occurred in Melbourne November 2016, the state government of Victoria introduced a range of measures to reduce the health impacts of future events (IGEM, 2017). While the exact mechanisms behind thunderstorm asthma are still not fully understood, such events are strongly associated with elevated grass pollen levels and specific weather conditions. To support risk prediction, a matrix was developed that combines forecasts of severe weather with district-level grass pollen forecasts, the latter produced and validated using data from a statewide pollen monitoring network (Bannister et al., 2021). Since 2017, an ETSA risk forecasting system has operated daily during Victoria’s grass pollen season (October to December) and is used to inform communities and the health and emergency services in their planning and preparation to ensure adequate response capacity.

As has been the case elsewhere in the world, pollen monitoring in Victoria is carried out by trained technicians who use microscopes to identify pollen in air samples collected by Hirst-type pollen and spore traps (Hirst, 1952; Ong et al., 1995). While these traps are considered the benchmark for pollen surveillance, their operation is labour-intensive and subject to well-recognised limitations, including variable sampling efficiency and human error in pollen identification and counting (Adamov et al., 2024). Together, these factors contribute to a commonly cited uncertainty of approximately ± 30% in daily pollen levels (Maya-Manzano et al., 2023).

Recent technological advances have led to the development of automated pollen counters that promise increased efficiency and reproducibility over the current manual methods (Buters et al., 2024; Maya-Manzano et al., 2023). Here, we compare pollen measurements from an automated pollen monitor, the SwisensPoleno Mars, with data from a co-located Hirst-type trap. Daily and higher-resolution measurements were evaluated with respect to data availability and the strength of any relationship between the two measurement systems. To facilitate comparison with previous studies, the analytical approach followed that of Maya-Manzano et al. (2023). Through this comparison, we aim to provide insights into the potential benefits and limitations of integrating automated systems into the existing pollen monitoring and ETSA risk forecasting infrastructure in Victoria.

## Materials and methods

### Trial site

Melbourne, Australia, is the state capital of Victoria. The trial site was co-located on the roof of the Earth Sciences building at the University of Melbourne, approximately 2 km north of the Central Business District (144.965°E, 37.797°S; 52 m ASL). This site became part of the statewide pollen monitoring network in 2017 but has been used for seasonal monitoring since 2003. Positioned 28.4 m above ground level (AGL), it complies with the AS/NZS 3580.1.1 standard for siting ambient air sampling units (Standards Australia, Sydney, NSW) and also meets the > 10 m AGL guideline for representative ambient pollen sampling (Rojo et al., 2019).

The trial commenced on October 1, 2022, and concluded on December 31, 2022, covering a 92-day period of the southern hemisphere spring and early summer. Over the 3 months of the trial Melbourne had 37 days with 1 mm or more of rain and received a total of 265.3 mm of rainfall. The maximum daily temperature over this period was 37 °C on December 27 and the minimum daily temperature was 5.2 °C on October 28.

### Burkard spore trap and manual counting

The Burkard spore trap (BSpo) is a Hirst-type volumetric pollen and spore trap (Burkard Manufacturing, Rickmansworth, England), (Hirst, 1952). Pollen grains are drawn in through a narrow inlet by a constant intake of air at 10 l min^−1^ and trapped on a standard glass microscope slide coated with an adhesive (Dow Corning Sylgard 527 silicone dielectric gel). At 9 am (AEDT) each day of the sampling period, the slide, attached to a rotating drum on the sampling head, was removed and replaced with a fresh slide. Daylight saving time (AEDT) began in Melbourne on 2 October 2022, but did not affect the time of sampling for the reason noted below.

The used was stained with Calberla’s stain and four longitudinal transects scanned at × 400 magnification to identify total, grass, and cypress pollen. The area counted was > 10% of the total to comply with the recommendations of Galán et al. (2014) and values were converted to concentrations (grains/m^3^) as described by Beggs et al. (2018). BSpo data was produced independently of the automated pollen dataset.

For sub-daily (3-hourly) pollen levels, daily slides were partitioned along their length into 8 × 6 mm regions with each region corresponding to a 3-h period starting at 9am. Three transverse transects were scanned in each region to identify pollen and the area counted was > 10% of the total.

### SwisensPoleno Mars and pollen classifier software

The SwisensPoleno Mars (PMars) is a flow cytometer that automatically detects, classifies, and counts airborne particles ranging in size from 2 to 300 µm in near real time. It continuously samples air at a flow rate of 40 L min^−1^, using light scattering to detect individual particles, and can report pollen concentrations at any desired temporal resolution. For each particle detected, two digital holograms are captured, allowing for the reconstruction of its cross-sectional shape at a resolution of 0.595 µm per pixel. These highly detailed reconstructions are analysed by an AI-based machine learning model (called MeteoSwiss 2022) that is trained to identify various types of pollen that are common in the air of Switzerland. The model can distinguish grass pollen (Poaceae) from pollen produced by several tree genera—*Alnus*, *Betula*, *Carpinus*, *Corylus*, *Cupressus*, *Fagus*, *Fraxinus*, *Pinaceae*, *Platanus*, *Populus*, *Quercus*, *Taxus*, and *Ulmus*—which are not native to Australia. (Sauvageat et al., 2020). All data were transmitted to an offsite server for storage.

This paper specifically assesses pollen concentrations for Poaceae (grasses), *Cupressus* (cypress), and *Taxus* (yew). Total pollen levels were estimated by summing the concentrations of all detected pollen types. Given that the MeteoSwiss 2022 classifier was trained on a predefined set of Swiss pollen classes, it was expected that pollen from Australian native plants would likely be misclassified and assigned with low probabilities across one or more existing classes rather than being recognised as a distinct pollen type. To enable comparison with manual counting methods, PMars data were adjusted using taxon-dependent scaling factors. These factors standardise automated measurements against corresponding manual counts to account for differences in particle capture and classification efficiency associated with the PMars system and the MeteoSwiss 2022 classifier. Scaling factors were applied separately to each pollen taxon and to total pollen concentrations.

### VGPEM model data

The Victorian Grass Pollen Emissions Module (VGPEM) is a quantitative model of grass pollen emissions and dispersion in Australia (Emmerson et al., 2019). Whole grass pollen levels were modelled for the University of Melbourne site using the physical characteristics of ryegrass pollen, the dominant grass pollen type in Melbourne’s atmosphere (Boyte et al., 2026), assuming a pollen grain diameter of 35 µm and a mass of 22.4 × 10⁻⁹ g. This representation also assumes that pollen grains are spherical with a unit density, ensuring a fall speed of 4.6 cm s^−1^ in the atmosphere. Emission estimates were based on a statistical configuration combining historical grass pollen measurements with enhanced vegetation index (EVI) data. Broadly, grass pollen is emitted when the EVI decreases by 5% of its winter maximum, and increases with higher temperatures, low humidity, and no rainfall. The model was run from 1 November 2022 to 18 January 2023, using meteorological inputs from ERA5 observations. These inputs were downscaled via the Conformal Cubic Atmospheric Model to 3 km spatial resolution and 1-h temporal resolution.

### Statistical analysis

Statistical tests were conducted using Microsoft Excel. For total, grass and cypress pollen, coefficients of determination (*R*^2^) were calculated to assess the agreement between the two monitoring systems by quantifying the strength of the linear relationship between their daily and sub-daily (3-hourly) pollen concentrations. Linear regression was performed to determine the slope of the relationship, with a slope close to 1.0 indicating similar performance between the two methods. For linear regression the fitted lines were forced to go through the origin point (0,0). Mean absolute error (MAE) was calculated to measure the average absolute differences between the values. The Wilcoxon signed-rank test was used to compare paired observations, ensuring robustness against non-normal data distributions.

For ETSA risk forecasting, daily grass pollen levels are not reported publicly as raw numerical values. Instead, they are reported in three operational categories used by the Victorian forecasting system: low (0–19 grains m^−3^), moderate (20–49 grains m^−3^), and high (≥ 50 grains m^−3^). These categories are central to public health decision-making because they form part of the inputs to the ETSA risk matrix and are used in public advisories. To evaluate whether the PMars and BSpo measurements fell into the same category on a given day, the Gerrity skill score was used (Gerrity, 1992). This score assesses the accuracy of categorical agreement relative to what would be expected by chance and was calculated using tools available at http://verification.tools/tools.

## Results

### General aspects of the trial and pollen taxa considered

**Fig. 1:**
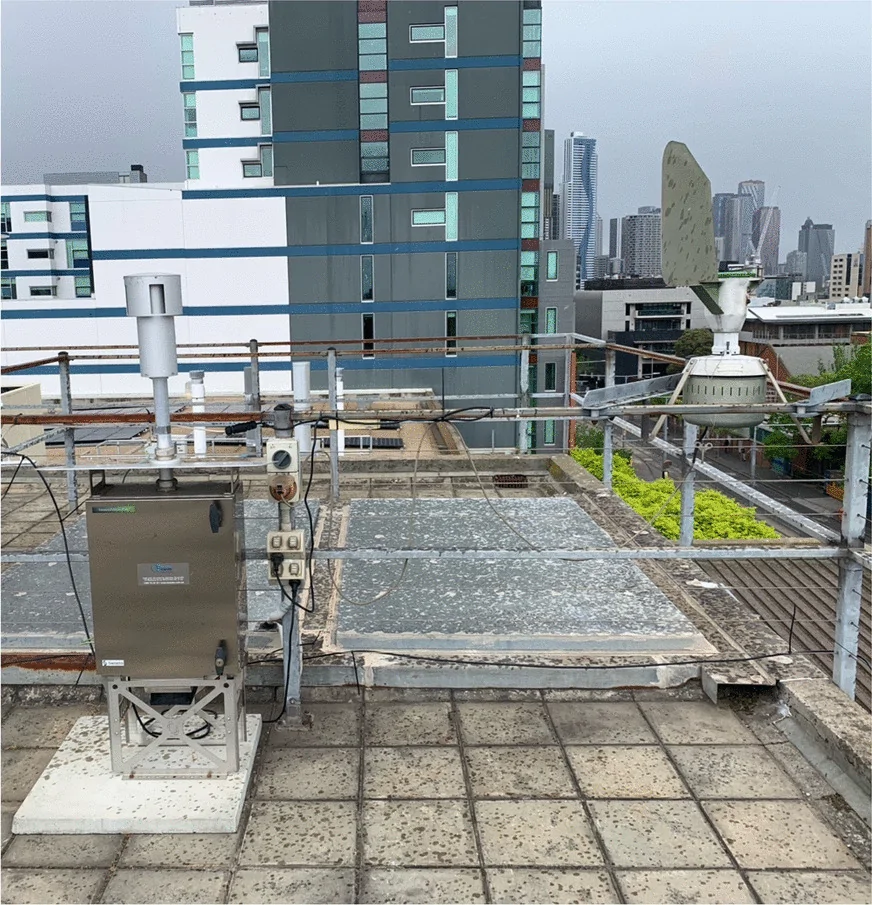
Site of the instrument comparison on the roof of the McCoy building at the University of Melbourne. The SwisensPoleno Mars (PMars) is on the left and the Burkard spore trap (BSpo) is on the right

Figure 1 shows the site used for the Melbourne trial. On the right is a Burkard Hirst spore (BSpo) trap, the manual instrument used as the reference instrument in this study. On the left is the SwisensPoleno Mars (PMars), an automated pollen counter that identifies selected pollen types (Sauvageat et al., 2020). The Melbourne PMars instrument used the MeteoSwiss 2022 classifier for grasses (Poaceae) and trees that are not native to Australia such as the evergreen conifers *Cupressus* (cypress) and *Taxus* (yew), as well as several deciduous trees. Data from the PMars was available for the entire duration of the trial (October 1 to December 31) but no data was available from the BSpo for October 1–5. Consequently, as noted in Table 1, a total of 88 days of BSpo data were available for comparison, with the change to AEDT occurring before BSpo monitoring began. The analytical methodology of Maya-Manzano et al. (2023) was followed to enable direct comparison with that study. PMars and reference BSpo pollen concentrations were compared at both daily and sub-daily (3-hourly) resolutions. Agreement between the two systems was assessed using the coefficient of determination (*R*^2^) to quantify the strength of the linear relationship, the linear regression slope to evaluate proportional bias, and the mean absolute error (MAE) to quantify the average prediction error relative to the BSpo measurements.

**Table 1:** Summary of pollen recorded by the BSpo instrument at the Melbourne site during the 2022 monitoring period (October 5–December 31)

	Total	Grass	Cypress
Number of reporting days	88	88	88
Average pollen level	101	37	32
Maximum daily level	1281	220	1148
Total pollen integral	8882	3218	2839

Total, grass and cypress pollen levels were monitored using the BSpo for comparison with PMars data. As PMars does not deliver total pollen concentrations, the sum of all identified pollen types was used instead. Grass and cypress were chosen because these are the predominant pollen types in Melbourne during the southern hemisphere spring and summer (Haberle et al., 2014).

Supplemental Figure S1 shows manually identified grass and cypress pollen grains along with the features used for their identification. While the main features of these grains remained consistent throughout the campaign, both pollen taxa exhibited variable morphologies, ranging from intact grains to broken grains with split walls and/or grains lacking cytoplasmic contents. All morphologies were included in the BSpo dataset.

Table 1 shows summary measures of pollen levels recorded by the BSpo during the campaign. Together, grass and cypress pollen made up nearly 70% of the total pollen integral. A notable feature was that around 40% of the total cypress pollen integral for the trial was recorded on the day with the highest level of this pollen type (October 12, 2022). This date was also the highest total pollen day and contributed around 14% to the total pollen integral.

The PMars system distinguishes between *Cupressus* and *Taxus* pollen, even though the two appear very similar and are often grouped into a single taxon in aerobiological studies (Tedeschini et al., 2021). While the presence of a pore can, in principle, be used to tell porate *Cupressus* grains from non-porate *Taxus* grains, this feature is difficult to see on intact grains and cannot be observed on split ones. *Taxus* pollen was also unlikely to be present in substantial amounts in Melbourne, where yew trees are relatively uncommon (e.g., see https://melbourneurbanforestvisual.com.au).

Given these factors, it was assumed that the classifier occasionally misidentified *Cupressus* grains as *Taxus*. To address this, all PMars *Taxus* counts were reassigned to *Cupressus* before comparison with manual cypress measurements. This adjustment yielded a ‘corrected *Cupressus’* value, calculated by applying the *Cupressus* scaling factor to the reported *Taxus* concentrations and then adding the adjusted *Taxus* values to the original *Cupressus* counts.

### Total pollen

**Fig. 2:**
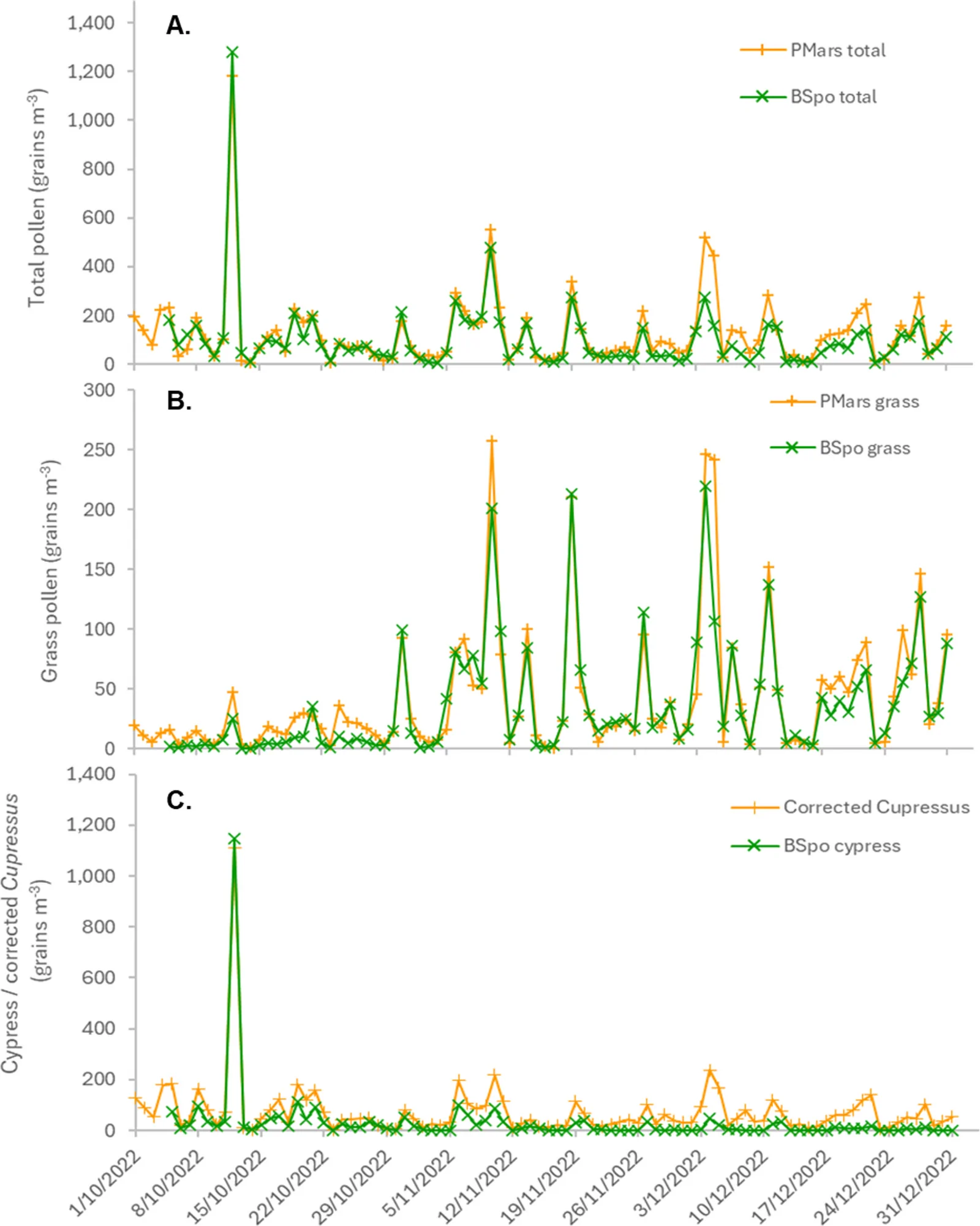
Seasonal profiles of (**A**) total pollen, **B** grass pollen and (**C**) corrected *Cupressus*/cypress pollen measured at the Melbourne trial site by the PMars and BSpo instruments

Figure 2A shows the seasonal profiles of total pollen measured by PMars and BSpo. The dominant peak on the left is the October 12 outlier mentioned above. Overall, the two sets of data showed good agreement, with manual counts tending to be lower than the automated ones, particularly toward the end of the monitoring period. As a result, the PMars recorded a total pollen integral that was 25% higher than the BSpo integral, even though this instrument had no classifier for some of the pollen types commonly seen in Melbourne’s air, such as those from the Myrtaceae family (eucalypts and bottlebrushes).

**Fig. 3:**
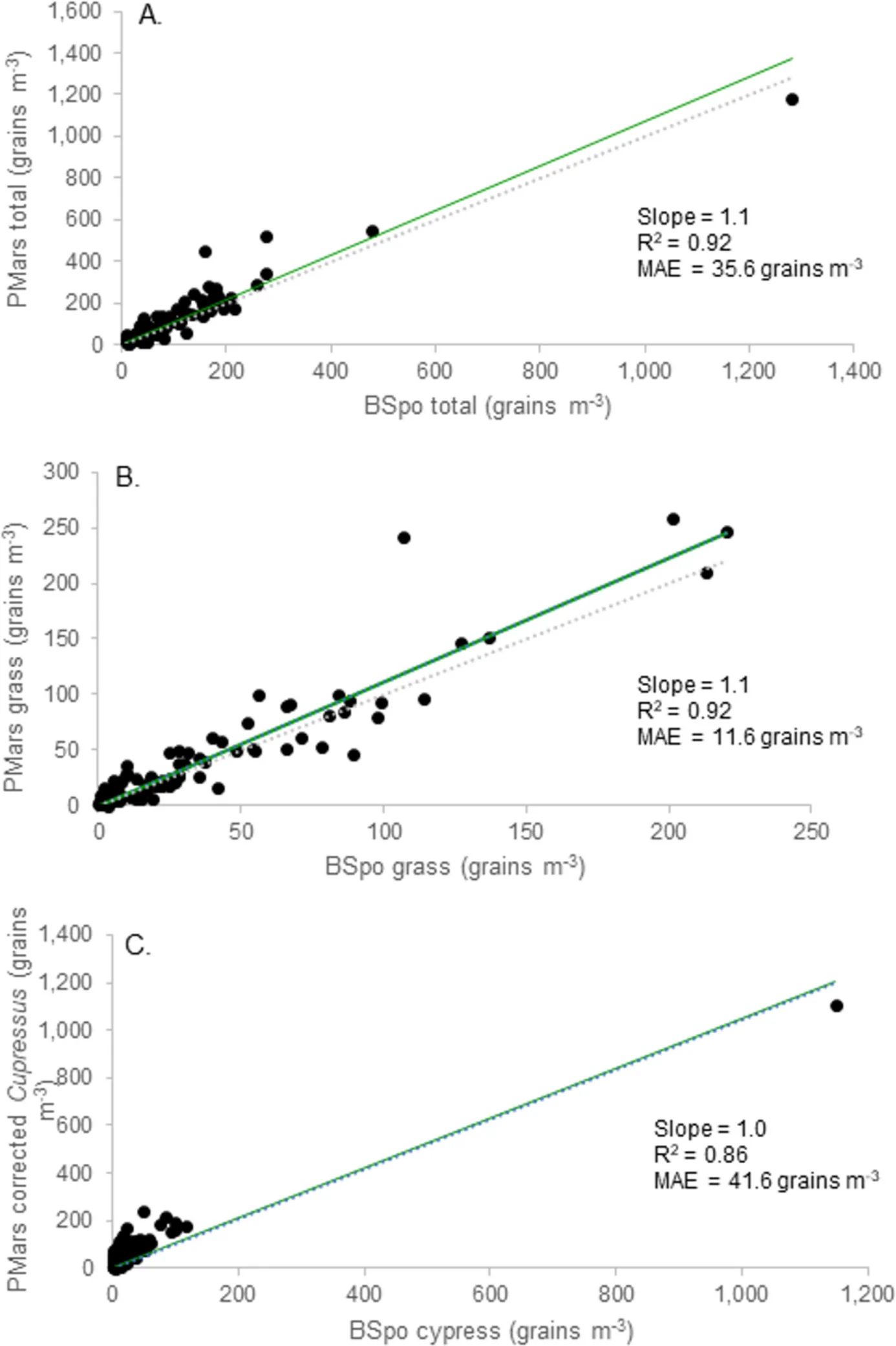
Daily scatter plots of the BSpo and PMars datasets for (**A**) total pollen, **B** grass pollen and (**C**) cypress/corrected *Cupressus* pollen. Green line is the line of best fit and the grey dashed line represents a perfect fit with the BSpo data. Slope, *R*^*2*^ and mean absolute error (MAE) values are also shown

Figure 3A shows a scatter plot comparing daily total pollen counts measured by PMars and BSpo, along with key statistical measures including the coefficient of determination (*R*^2^), slope of the line of best fit, and mean absolute error (MAE). Table 2 summarises these statistics and the results of a Wilcoxon signed-rank test (a non-parametric test to assess whether the differences between PMars and BSpo measurements are statistically significant), alongside the corresponding values reported by Maya-Manzano et al. (2023). Perfect agreement between PMars and BSpo measurements would yield *R*^2^ = 1.0, slope = 1.0, and MAE = 0.

**Table 2:** Daily and 3-hourly average statistical values (*R*^2^ and slope, mean absolute error (MAE), and Wilcoxon signed-rank test) for the PMars for total, grass, and cypress pollen

	Total						Poaceae		
	3-hourly			Daily*			3-hourly		
System	*R*^2^/slope	MAE	Wilcoxon signed-rank test (vs. BSpo)	*R*^2^/slope	MAE	Wilcoxon signed-rank test (vs. BSpo)	*R*^2^/slope	MAE	Wilcoxon signed-rank test (vs. BSpo)
Melbourne PMars	0.86/0.9	65.6	**0.24**	0.92/1.1	35.64	< 0.00001	0.81/0.9	27.8	**0.78**
Munich PMars (Maya-Manzano)	0.49/0.83	93.74	< 0.001	0.55/0.83	78.58	< 0.001	0.51/0.81	19.46	**0.25**

				Cypress					
	Daily			3-hourly			Daily*		
System	*R*^2^/slope	MAE	Wilcoxon signed-rank test (vs. BSpo)	*R*^2^/slope	MAE	Wilcoxon signed-rank test (vs. BSpo)	*R*^2^/slope	MAE	Wilcoxon signed-rank test (vs. BSpo)
Melbourne PMars	0.92/1.1	11.6	< 0.001	0.72/1.6	49.3	< 0.00001	0.86/1.0	41.6	< 0.00001
Munich PMars (Maya-Manzano)	0.82/0.85	12.75	**0.40**						

An asterisk indicates the daily cypress values include the October 12 outlier. All statistics are calculated compared to BSpo manual observations. Bold values indicate those that are not statistically significant different from the BSpo data (*p* > 0.05)

The line of best fit in Fig. 3A had an *R*^2^ = 0.92 and a slope of 1.1, which equates to excellent agreement between the PMars and BSpo total pollen observations. The slope is consistent with PMars providing slightly higher total pollen values. Table 2 shows that the correspondence between the PMars and BSpo total pollen measurements was better (larger *R*^2^, smaller MAE, similar slope) in Melbourne than had been reported for the Munich trial by Maya-Manzano et al. (2023). This is possibly because of the dominance of grass and cypress pollen during the monitoring period in Melbourne, both of which the PMars can identify.

Twelve days from around the season’s November midpoint were selected for sub-daily analysis, producing a dataset of 96 BSpo total pollen values at 3-hourly resolution. Across the 12 selected days, previously determined BSpo daily total pollen concentrations ranged from 13 to 477 pollen grains m^−3^, with an average of 127 grains m^−3^. About half the days had total daily pollen concentrations below 50 grains m^−3^. Supplemental Fig. S2A shows that 3-hourly pollen concentrations were generally higher during the day (3 h periods ending 12 noon to 9 pm) and lower overnight (12 midnight to 9 am) with 65–75% of the daily total recorded during the day.

**Fig. 4:**
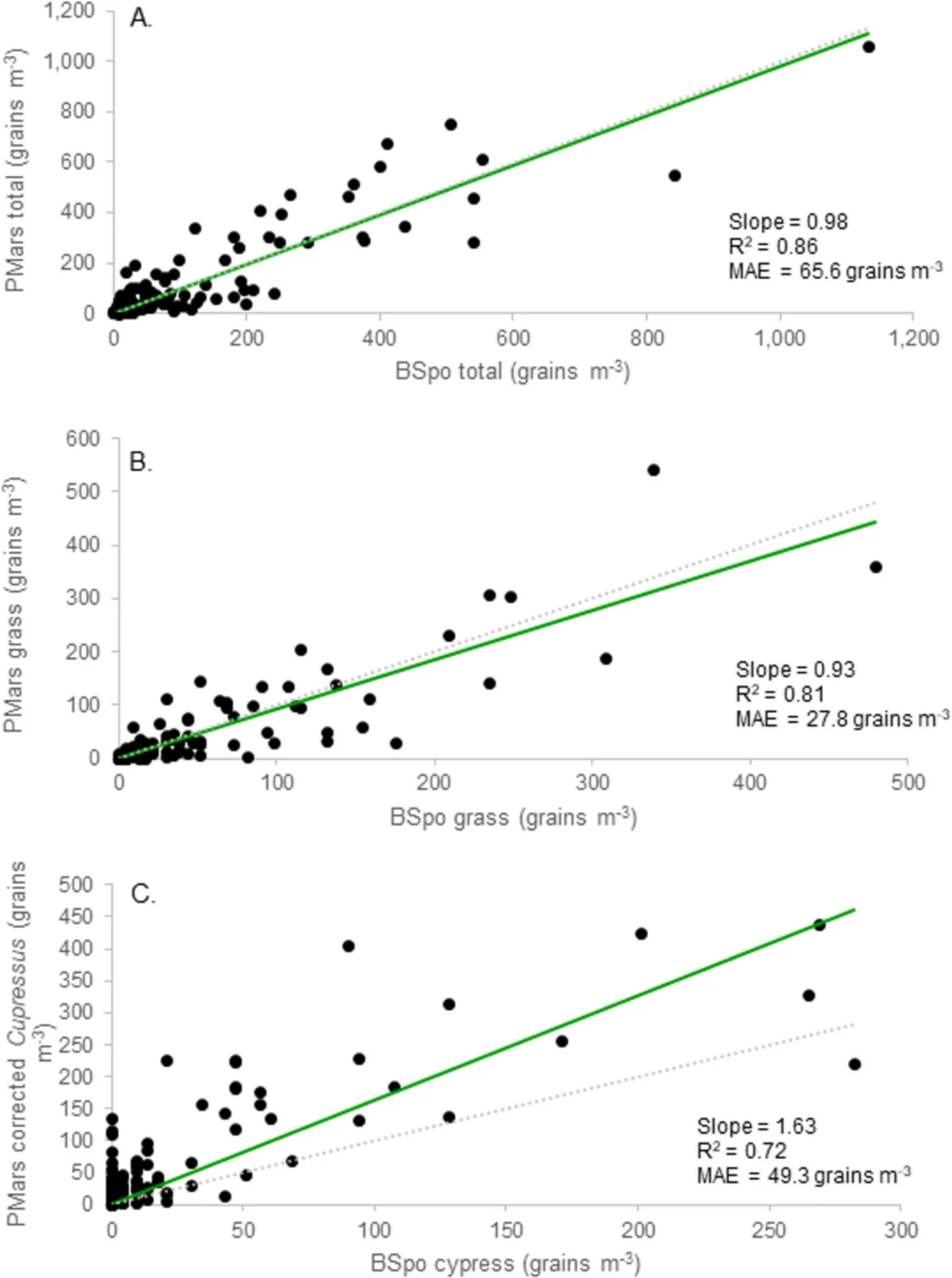
Three hourly resolved scatter plots for (**A**) total pollen, **B** grass pollen and (**C**) cypress/corrected *Cupressus* pollen measured by the BSpo and PMars. The green line is the line of best fit and the grey dashed line represents a perfect fit with the BSpo data. *R*^2^, slope of the line of best fit and MAE values are also shown

Figure 4A shows there was a strong linear association (*R*^2^ = 0.86) between the BSpo and PMars total pollen values. The slope (0.9) was similar to that observed for daily data (see Fig. 3A). Consistent with the findings of Maya-Manzano et al. (2023), MAE at 3-hourly resolution was larger than at daily resolution (65.6 vs 35.64 grains m^−3^; Table 2).

### Grass pollen

Like total pollen, the seasonal profile of grass pollen (Fig. 2B) showed strong association between the PMars and BSpo values with PMars daily values being generally slightly higher than BSpo values. Overall, the PMars grass pollen integral was 16% larger than the BSpo grass pollen integral.

Figure 3B shows a strong linear association between the BSpo and PMars daily grass pollen levels (*R*^2^ = 0.92, slope = 1.1 and MAE = 11.6 grains m^−3^). The values were consistent with previously reported values for the PMars/BSpo comparison of Maya-Manzano et al. (2023; Table 2).

**Fig. 5:**
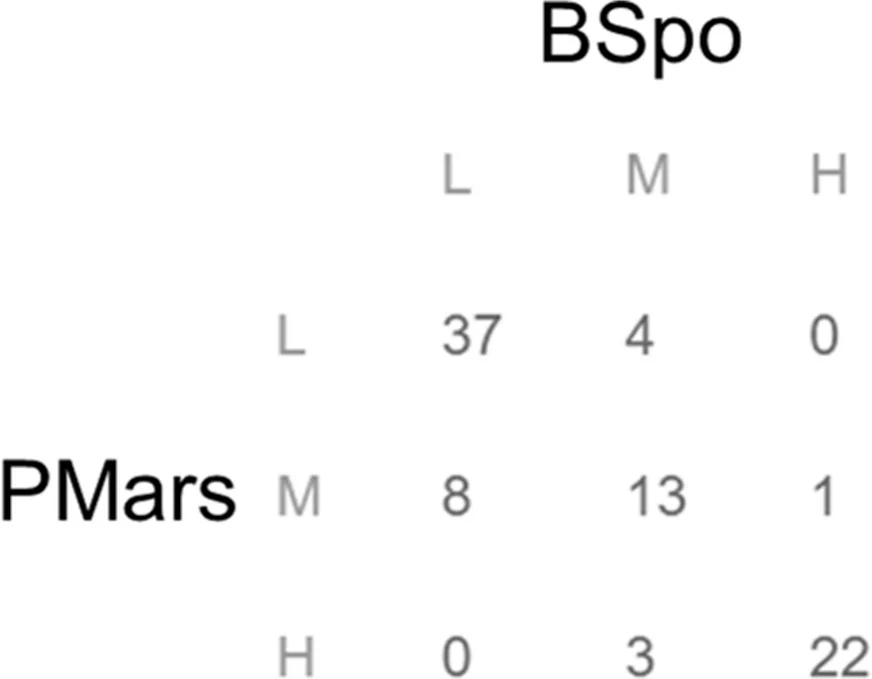
Confusion table of 88 pairs of daily grass pollen categories, low (L), moderate (M) and high (H), as determined by PMars and BSpo

In Victoria, thunderstorm asthma forecasts and public reports of daily grass pollen levels use categorical ratings (low, moderate, and high) rather than numerical values. To assess how closely PMars matched the BSpo classifications, the 88 paired daily readings were compared using a confusion matrix (Fig. 5). Of these, 72 pairs fell into the same category. Among the 16 mismatches, 12 were between the low and moderate categories, and four were between moderate and high. This tendency for greater disagreement at lower pollen categories is consistent with previous studies made using co-located BSpo traps, which found that instrument variability is highest when pollen concentrations are low and decreases as concentrations rise (Adamov et al., 2024). PMars correctly identified BSpo high-pollen days 96% of the time (probability of detection = 0.96) and achieved a Gerrity score of 0.82, indicating strong categorical agreement beyond chance. Notably, during the trial, PMars produced no false positives (low BSpo, high PMars) and no false negatives (high BSpo, low PMars). This is important because false positives can trigger unwarranted ETSA risk alerts, resulting in unnecessary costs for agencies, warning fatigue, and a loss of credibility that ultimately undermines the value of these warnings. Conversely, false negatives risk failing to issue alerts during genuine high-risk periods, potentially leaving susceptible individuals unprotected and increasing the likelihood of adverse health outcomes and strain on the health and emergency services.

Figure 4B shows that the strength of the linear relationship between BSpo and PMars grass pollen values was slightly weaker at 3-hourly resolution than at daily resolution (*R*^2^ = 0.81 vs 0.92), with a higher mean absolute error (MAE; 27.8 grains m^−3^ vs 11.6 grains m^−3^). However, the slopes of the lines of best fit were similar (0.9 vs 1.1). Supplemental Fig. S2B indicates that 3-hourly pollen concentrations were generally higher during the day and lower at night, a diurnal pattern previously reported by Mampage et al. (2025) using hourly PMars measurements.

The diurnal pattern observed here is also consistent with outputs from the Victorian Grass Pollen Emissions Module (VGPEM), a dispersion model that simulates the atmospheric transport of grass pollen from source regions (Emmerson et al., 2019). For the available dates, grass pollen levels predicted by VGPEM were closely associated with observed grass pollen concentrations, with *R*^*2*^ values of 0.69 for daily BSpo counts and 0.44 for hourly PMars data (Mampage et al., 2025). VGPEM provides an independent representation of the expected diurnal cycle in grass pollen, including increases associated with warming and drying conditions and decreases during cooler, more humid periods. Comparing observations with VGPEM therefore provided an external benchmark for assessing whether the instruments captured the expected sub-daily variation in grass pollen concentrations. For the 3-hourly data examined in this study, both instruments showed strong linear relationships with VGPEM outputs (PMars *R*^2^ = 0.58; BSpo *R*^2^ = 0.54; Supplemental Fig. S3), confirming the modelled day-to-night pattern, with pollen concentrations increasing through the middle of the day and declining overnight.

### Cypress pollen

Due to the difficulty distinguishing *Cupressus* and *Taxus* pollen manually, and because the MeteoSwiss 2022 classifier has separate classes for *Cupressus* and *Taxus*, BSpo cypress pollen data were compared to corrected PMars *Cupressus* data, which combined both the *Cupressus* and *Taxus* value concentrations while using the scaling factor for *Cupressus*.

Figure 2C shows the seasonal profiles of BSpo cypress pollen and corrected PMars *Cupressus*. The Oct 12 outlier is seen at the left of the figure. Good agreement was seen between the two profiles although BSpo cypress values are lower than corrected PMars *Cupressus*, especially towards the end of the season. Significant discrepancies between the BSpo and PMars values near the end of the trial period led to the corrected PMars *Cupressus* pollen integral being more than double the BSpo cypress pollen integral.

Figure 3C shows scatter plots of daily BSpo cypress measurements compared with corrected PMars *Cupressus* data. The two datasets showed a strong linear association (*R*^2^ = 0.86) with a slope of 1.0 and a MAE of 41.6, but these values were heavily influenced by the October 12 outlier. Excluding that point increased the slope to 2.2, while *R*^*2*^ (0.77) and MAE (41.6) changed little (see Supplemental Fig. S4C). The increase in slope was expected given the large difference between the PMars and BSpo cypress pollen integrals. In contrast, removing the outlier produced only small changes in the corresponding daily total pollen and grass pollen metrics (Supplemental Fig. S4A, B).

At the 3-h resolution, the line of best fit between the BSpo cypress and corrected PMars *Cupressus* values was steeper than the corresponding daily relationship (1.6 vs 1.0). However, because the daily slope was strongly influenced by the October 12 outlier, the outlier-excluded daily slope (2.2; Supplemental Fig. S4C) provides a more appropriate comparison. The 3-h values were also less strongly associated than the daily values (*R*^2^ = 0.72 vs 0.86) and had a higher MAE (49.3 vs 41.6 grains m^−3^; Fig. 4C). These differences mirror those observed between daily and 3-h total pollen, as well as daily and 3-h grass pollen.

## Discussion

This trial showed that the SwisensPoleno Mars (PMars) performed well in Melbourne’s aerobiological environment, generating total, grass, and cypress pollen measurements that showed strong linear associations with those from the manual Burkard Hirst spore trap (BSpo) at both daily and sub-daily scales. These results indicate that PMars reliably captured temporal variation in airborne pollen concentrations, including during high-load events such as the 12 October outlier day. These findings are consistent with recent evaluations of automated pollen monitors, which have shown that instruments like the PMars can provide all the functions of traditional Hirst traps while producing data streams that are suitable for operational use in near real time (Farooq et al., 2025).

PMars performance for grass pollen, the most important pollen type for public health in Victoria, was particularly encouraging. Daily PMars grass counts closely matched BSpo values, and categorical agreement across the low, moderate, and high thresholds used in thunderstorm asthma forecasting was high, with no false positives or false negatives for high pollen days. Sub-daily comparisons also showed good correspondence and reproduced expected diurnal cycles consistent with the VGPEM dispersion model predictions. The ability to produce reliable sub-daily pollen data is increasingly recognised as important for understanding rapid changes in pollen exposure, particularly during thunderstorm asthma events, and aligns with moves towards higher temporal resolution in operational pollen services in other countries (Tummon et al., 2024). Together, these results indicate that the PMars is well suited for operational grass-pollen monitoring in support of public health operations, and with the potential to be employed in future research into the mechanisms of thunderstorm asthma, especially the ease with which sub-daily data can be generated.

Cypress pollen presented a more complex case because the PMars classifier differentiated what is likely a single pollen type into *Cupressus* and *Taxus*. After correction, PMars *Cupressus* values showed strong linear relationships with BSpo cypress counts at both daily and 3-hourly resolutions. However, substantial discrepancies in seasonal integrals and sensitivity to the extreme October 12 event highlight the need for classifier refinement and further evaluation of Cupressaceae detection by automated systems. Similar challenges have been reported in other automated pollen monitoring trials, where classifier performance for specific taxa varies depending on region and training data, reinforcing the need for locally adapted models (Tummon et al., 2024). Importantly, these issues were largely confined to cypress and did not materially affect comparisons for grass or total pollen.

## Study limitations

The co-location of the SwisensPoleno Mars and Burkard spore trap provided a strong basis for evaluating automated pollen monitoring under real operational conditions. Both instruments sampled the same rooftop environment over the same spring monitoring period, enabling direct comparison of daily and sub-daily pollen measurements at an established Melbourne monitoring site. As with any field evaluation, the extent to which these results generalise will depend on the pollen assemblages, meteorological conditions and background particle environments encountered in other regions. Further evaluations across additional Australian sites and seasons would help define performance under a broader range of vegetation settings, including areas where native pollen taxa contribute more substantially to the airborne pollen load.

The classifier configuration also needs to be considered when interpreting taxon-specific results. The SwisensPoleno Mars was operated with the MeteoSwiss 2022 classifier, which was developed using European pollen training data rather than a classifier optimised for Australian aerobiological conditions. This provided a useful test of the instrument in a new operational setting, but also explains why some taxonomic categories required local interpretation. The reassignment of *Taxus* counts to *Cupressus* was based on the biological similarity of these pollen types, their established grouping in many aerobiological studies, and the low likelihood of substantial *Taxus* pollen contributions in Melbourne. The strong agreement observed for grass pollen is therefore particularly important, because grass is the priority taxon for thunderstorm asthma forecasting in Victoria. The cypress results should be interpreted mainly as evidence that local classifier refinement will improve taxonomic resolution for some non-grass taxa.

The Burkard spore trap was used as the reference method because Hirst-type volumetric traps remain the operational benchmark for pollen monitoring in Victoria and internationally. However, manual pollen counting is itself subject to sampling, preparation and counting uncertainty. Agreement with the Burkard trap therefore evaluates operational comparability with the current monitoring standard rather than agreement with an absolute particle-level ground truth. This is appropriate for the present study because existing Victorian pollen records, public reporting categories and thunderstorm asthma forecasting thresholds are based on Hirst-type monitoring data.

Finally, the sub-daily analysis used a focused set of paired 3-hourly observations. These data were sufficient to test whether the automated system could reproduce short-term temporal structure in pollen concentrations, including expected day-to-night variation, but larger multi-season datasets will be needed to quantify performance across rarer combinations of high pollen, rainfall, humidity and storm-related meteorological conditions. The study also did not directly link automated pollen measurements to health outcomes. Instead, it evaluated the exposure-measurement component that underpins public health forecasting. Future work linking automated pollen data with syndromic surveillance, ambulance call-outs, emergency department presentations or asthma admissions would help quantify the additional public health value of higher-resolution pollen monitoring.

## Conclusions

The Melbourne trial demonstrated that the SwisensPoleno Mars produces near-real-time pollen measurements that correspond closely with traditional manual measurements at both daily and sub-daily scales. Agreement was particularly strong for grass pollen, the pollen type most relevant to thunderstorm asthma forecasting in Victoria, with strong correspondence between the two systems for daily concentrations and for the operational low, moderate and high pollen categories. The automated system also reproduced expected sub-daily variation in pollen concentrations, demonstrating its ability to capture changes that are not resolved by routine daily manual counting. Cypress pollen classification was more complex and would benefit from further refinement of the classifier for Australian conditions. Overall, the principal added value of automated monitoring is the ability to provide reliable pollen measurements at substantially higher temporal resolution while reducing reliance on labour-intensive manual counting. These findings support the integration of automated pollen counters into routine aerobiological monitoring and public health forecasting systems in Australia.

## Supplementary Information

Below is the link to the electronic supplementary material.

Supplementary file1 (DOCX 2.03 MB)

## Data Availability

The data that support the findings of this study are available from AirHealth Pty Ltd but restrictions apply to the availability of these data, which were used under license for the current study, and so are not publicly available. Data are however available from the authors upon reasonable request and with permission of AirHealth Pty Ltd.
