# Supplementary material for: Evaluating automated pollen monitoring for thunderstorm asthma and public health forecasting: a comparative study in Melbourne, Australia
Source: Environ Monit Assess. 2026 Oct 13;198(11):1187. doi: 10.1007/s10661-026-16017-2 (PMC13661651; doi:10.1007/s10661-026-16017-2)
Supplement: Supplementary file 1 [file 10661_2026_16017_MOESM1_ESM.docx]

Evaluating Automated Pollen Monitoring for Thunderstorm Asthma and Public Health Forecasting: A Comparative Study in Melbourne, Australia

Edwin R. Lampugnani^1,2,3,4^, Usha Nattala^2,5^, Danny Csutoros^6^, Kale Sniderman^7^, Beau Picking^2^, Kathryn M. Emmerson^8^, Elias Graf^9^, Yanick Zeder^9^, Erny Niederberger^9^ and Ed Newbigin^1,2^

1. Department of Medicine (RMH), University of Melbourne, Parkville, VIC 3010, Australia
2. AirHealth Pty Ltd., PO Box 38 Parkville, VIC 3052, Australia
3. School of Health Sciences, University of Melbourne, Parkville, VIC 3010, Australia
4. Menzies Institute for Medical Research, College of Health and Medicine, University of Tasmania, Hobart, TAS 7001, Australia
5. Melbourne Data Analytics Platform, University of Melbourne, Parkville VIC 3010 Australia
6. Victorian Department of Health, Melbourne VIC 3000 Australia
7. School of Geography, Earth and Atmospheric Sciences, University of Melbourne, Parkville, VIC 3010, Australia
8. CSIRO Environment, Aspendale, VIC 3195, Australia
9. Swisens AG, Meierhofstrasse 5a, 6032 Emmen, Switzerland

# Supporting Information

Supplemental Figure 1: Light microscope images of pollen grains treated with Calberla’s stain that were identified as cypress (A-E) and grass (F-H). Intact cypress pollen grains (A-C) were approximately 30μm in diameter, spherical, psilate with unevenly distributed verrucae and a prominent polygonal inclusion or “nucleus” *sensu* Duhoux (1982) (arrow in A). One (B) or more (C) possible circular pores were observed on some intact cypress pollen grains (arrows). Split cypress pollen grains (D-E) typically displayed a “Pacman-like” appearance. Split cypress pollen grains (D) occasionally had an attached cytoplasm (c), polygonal “nucleus” (n) and dilated intine (i). Clumps of cypress pollen grains (E) with a range of these morphologies were sometimes observed. Intact grass pollen grains (F) were approximately 30-40μm in length, spheroidal or ovoid in shape, with a granular cytoplasm and a single pore surrounded by an annulus (arrow). Grass pollen grains were also observed with partial (G) or complete loss (H) of cellular contents. G shows a grass pollen grain with partial loss of cellular contents (arrow). H shows the pore (arrow) of a grass pollen grain that lacked cellular contents. Bar scale indicates size.

Supplemental Figure 2: Box plots showing 3-hourly resolved plots for A) total pollen, B) grass pollen and C) cypress/corrected *Cupressus* pollen as measured by the BSpo and PMars, each normalised to their respective daily totals. B) also shows the normalised 3-hourly resolved grass pollen outputs from VGPEM (see text). The x-axis indicates the end time of each 3-hour interval, and lines connect the mean values.

Supplemental Figure 3: Three hourly resolved scatter plots comparing A) PMars grass pollen and B) BSpo grass pollen with three hourly average VGPEM outputs. Green line is the line of best fit and the grey dashed line represents a perfect fit with either the PMars or BSpo data. R^2^, slope of the line of best fit and MAE values are also shown.

Supplemental Figure 4: Daily scatter plots without the October 12 outlier of the Burkard spore trap (BSpo) and Poleno Mars (PMars) datasets for A) total pollen, B) grass pollen and C) cypress/corrected *Cupressus* pollen. Green line is the line of best fit and the grey dashed line represents a perfect fit with the BSpo data. Slope, R^2^ and mean absolute error (MAE) are also shown.

**References**

Duhoux, E. (1982) Mechanism of exine rupture in hydrated taxoid type pollen. *Grana* **21**, 1-7. <https://doi.org/10.1080/00173138209427673>
